# Benzyl *N*-((*S*)-2-hydr­oxy-1-{*N*′-[(*E*)-2-methoxy­benzyl­idene]hydrazinecarbon­yl}eth­yl)carbamate from synchrotron data

**DOI:** 10.1107/S1600536810011463

**Published:** 2010-03-31

**Authors:** Alessandra C. Pinheiro, Marcus V. N. de Souza, Edward R. T. Tiekink, James L. Wardell, Solange M. S. V. Wardell

**Affiliations:** a Instituto de Tecnologia em Farmacos, Fundação Oswaldo Cruz (FIOCRUZ), FarManguinhos, Rua Sizenando Nabuco, 100, Manguinhos, 21041-250 Rio de Janeiro, RJ, Brazil; b Department of Chemistry, University of Malaya, 50603 Kuala Lumpur, Malaysia; c Centro de Desenvolvimento Tecnológico em Saúde (CDTS), Fundação Oswaldo Cruz (FIOCRUZ), Casa Amarela, Campus de Manguinhos, Av. Brasil 4365, 21040-900 Rio de Janeiro, RJ, Brazil; d CHEMSOL, 1 Harcourt Road, Aberdeen AB15 5NY, Scotland

## Abstract

A U-shaped conformation is found in the title compound, C_19_H_21_N_3_O_5_, with the benzene rings lying to the same side of the mol­ecule; the dihedral angle between them is 10.83 (16)°. The dihedral angle formed between the hydrazinecarbonyl and carbamate residues is 68.42 (13)°. The carbonyl groups lie approximately at right angles to each other [O—C⋯C—O pseudo torsion angle of 107.7 (3)°], and the conformation about the C12=N3 bond [1.279 (4) Å] is *E*. An intra­molecular N_cb_—H⋯O_hy_ (cb = carbmate and hy = hydr­oxy) hydrogen bond occurs, generating an *S*(6) loop. In the crystal, inter­molecular O_h_—H⋯O_ca_ (ca = carbon­yl) and N_hz_—H⋯O_ca_ (hz = hydrazine) hydrogen bonds lead to the formation of a supra­molecular chain, two mol­ecules thick, which propagates along the *a* axis; these are connected by C—H⋯O_ca_ contacts.

## Related literature

For background to tuberculosis, see: Cole & Alzari (2007 ▶); Portero & Rubio (2007 ▶). For information on the development of anti-tuberculosis agents, see: Lourenço *et al.* (2007*a* ▶,*b* ▶); Lourenço *et al.* (2008 ▶); Ferreira *et al.* (2008 ▶); Costa *et al.* (2006 ▶); de Souza *et al.* (2006*a* ▶,*b* ▶); Pinheiro *et al.* (2007 ▶).
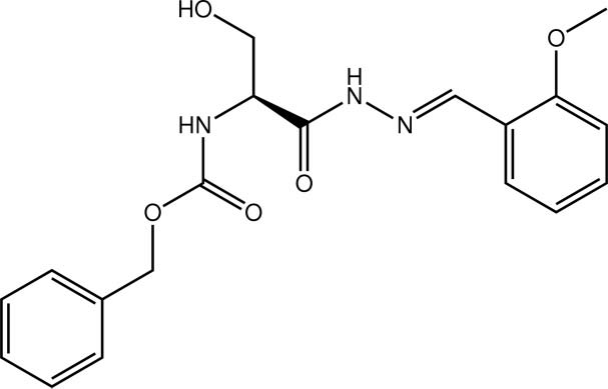


## Experimental

### 

#### Crystal data

C_19_H_21_N_3_O_5_
*M* *_r_* = 371.39
Orthorhombic, 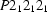

*a* = 6.002 (6) Å
*b* = 14.053 (14) Å
*c* = 21.09 (2) Å
*V* = 1779 (3) Å^3^
*Z* = 4
Synchrotron radiation
λ = 0.6889 Å
μ = 0.06 mm^−1^
*T* = 120 K
0.30 × 0.04 × 0.02 mm

#### Data collection

Rigaku Saturn 724+ detector on Crystal Logics CCD diffractometer
13639 measured reflections
1827 independent reflections
1627 reflections with *I* > 2σ(*I*)
*R*_int_ = 0.047

#### Refinement

*R*[*F* ^2^ > 2σ(*F* ^2^)] = 0.030
*wR*(*F* ^2^) = 0.124
*S* = 1.29
1827 reflections
257 parameters
3 restraints
H atoms treated by a mixture of independent and constrained refinement
Δρ_max_ = 0.22 e Å^−3^
Δρ_min_ = −0.31 e Å^−3^

### 

Data collection: *CrystalClear* (Rigaku/MSC, 2008 ▶); cell refinement: *APEX2* (Bruker, 2008 ▶); data reduction: *APEX2*; program(s) used to solve structure: *SHELXS97* (Sheldrick, 2008 ▶); program(s) used to refine structure: *SHELXL97* (Sheldrick, 2008 ▶); molecular graphics: *ORTEP-3* (Farrugia, 1997 ▶) and *DIAMOND* (Brandenburg, 2006 ▶); software used to prepare material for publication: *publCIF* (Westrip, 2010 ▶).

## Supplementary Material

Crystal structure: contains datablocks global, I. DOI: 10.1107/S1600536810011463/hb5379sup1.cif

Structure factors: contains datablocks I. DOI: 10.1107/S1600536810011463/hb5379Isup2.hkl

Additional supplementary materials:  crystallographic information; 3D view; checkCIF report

## Figures and Tables

**Table 1:** Hydrogen-bond geometry (Å, °)

*D*—H⋯*A*	*D*—H	H⋯*A*	*D*⋯*A*	*D*—H⋯*A*
N1—H1n⋯O3	0.89 (3)	2.44 (3)	2.788 (5)	104 (2)
O3—H3o⋯O4^i^	0.85 (2)	2.04 (2)	2.789 (4)	147 (4)
N2—H2n⋯O2^ii^	0.88 (3)	2.10 (3)	2.974 (5)	177 (3)
C10—H10b⋯O2^i^	0.99	2.45	3.439 (5)	175
C16—H16⋯O4^iii^	0.95	2.55	3.284 (5)	134

Symmetry codes: (i) 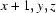; (ii) 
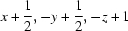
; (iii) 
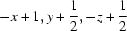
.

**Fig. 1.**
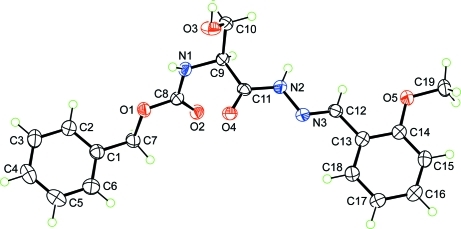
The molecular structure of (I) showing displacement ellipsoids at the 50% probability level.

**Fig. 2.**
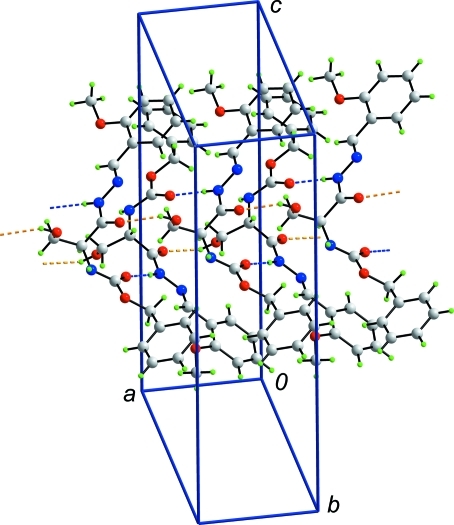
The supramolecular double-chain aligned along the a axis in the crystal structure of (I) formed through the agency of intermolecular O–H···O and N–H···O hydrogen bonding interactions shown as orange and blue dashed lines, respectively.

**Fig. 3.**
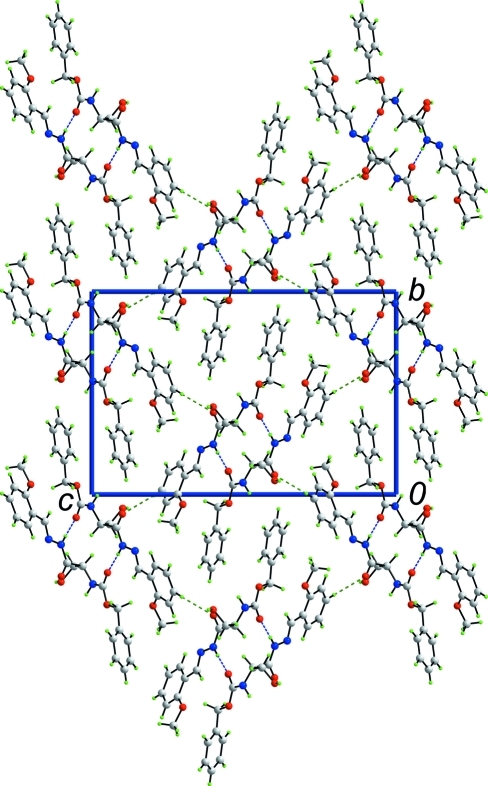
A view of the unit cell contents in (I) shown in projection down the a axis [the direction of the supramolecular chains illustrated in Fig. 2] and highlighting the C–H···O interactions (green dashed lines) formed between the double-chains; N–H···O interactions are shown as blue dashed lines.

## supplementary crystallographic information

### Comment

Tuberculosis (TB) is once again a major public health problem. The need for better drugs is clear (Cole & Alzari, 2007; Portero & Rubio, 2007). Continuing our studies on potential anti-tuberculosis agents (Lourenço *et al.*, 2007*a*, 2007*b*, 2008; Ferreira *et al.*, 2008; Costa *et al.*, 2006; de Souza *et al.*, 2006*a*, 2006*b*), including serinyl derivatives (Pinheiro *et al.*, 2007), we have investigated a series of serinyl derivatives, *N*-(2-hydroxy-1-{*N*'-[(1*E*)-(2- methoxyphenyl)methylidene]hydrazinecarbonyl}ethyl)carbamic acid esters and now report the structure of one of these, benzyl *N*-(2-hydroxy-1-{*N*'-[(1*E*)-(2- methoxyphenyl)methylidene] hydrazinecarbonyl}ethyl)carbamate, (I).

Overall, the molecule of (I), Fig. 1, has a U-shaped conformation with the benzene rings lying to the same side of the molecule; the dihedral angle between their least-squares planes is 10.83 (16) °. The dihedral angle formed between the least-squares planes through the hydrazinecarbonyl (r.m.s. deviation of the O4,N2,N3,C11 atoms = 0.0198 Å) and carbamate (r.m.s. deviation of the O1,O2,N1,C8 atoms = 0.0044 Å) is 68.42 (13) °. The carbonyl groups are approximately at right-angles to each other as seen in the pseudo O2–C8···C11–O4 torsion angle of 107.7 (3) °. Each of the N–H groups is *anti* to the adjacent carbonyl so that the N–H groups also lie to opposite sides of the molecule. The conformation about the C12═N3 bond [1.279 (4) Å] is *E*. Although the absolute structure could not be determined experimentally, the assignment of the *S*-configuration at the C2 atom is based on a starting reagent.

There are three acidic H atoms in the molecule of (I) and each of these forms a significant hydrogen bond, Table 1. Whereas the carbamate-N1–H atom forms an intramolecular N–H···O hydrogen bond to the hydroxyl-O3 atom, the hydroxyl-O3–H atom forms an intermolecular O–H···O interaction with the carbonyl-O4 atom, and the hydrazine-N2–H atom likewise forms an N–H···O hydrogen bond with the carbonyl-O2 atom. The intermolecular hydrogen bonds lead to the formation of a supramolecular double-chain along the *a* direction, Fig. 2, with additional stability to the chain afforded by C–H···O interactions involving the carbonyl-O2 atom, Table 1. The primary interactions between chains are of the type C–H···O and involve the carbonyl-O4 atom, Table 1 and Fig. 3.

### Experimental

The compound, phenyl (1*S*)-2-hydrazino-1-(hydroxymethyl)-2-oxoethylcarbamate, was obtained from L-serine methyl ester hydrochloride on successive reactions with (a) PhCH_2_Cl and Et_3_N, and (b) N_2_H_4_.H_2_O. To a stirred ethanol solution (10 ml) of phenyl (1*S*)-2-hydrazino-1-(hydroxymethyl)-2-oxoethylcarbamate (1.0 mmol), at room temperature was added 2-methoxybenzaldehyde (1.05 mmol). The reaction mixture was stirred for 4 hours at 353 K and concentrated under reduced pressure. The residue was purified by washing with cold ethanol (3 × 10 ml), affording the title compound; yield 80%. Solution NMR spectra revealed the presence of (*E*)- and (*Z*)-isomers, however, the colourless needles of (I) obtained from MeOH solution were solely the (*E*)-isomer, m.pt. 453–454 K.

^1^H NMR (400 MHz, DMSO-d_6_) δ (ppm): 11.48 and 11.36 (1*H*, s, NHN, *E*- and *Z*- isomers), 8.59 and 8.33 (1*H*, s, N=CH, *E*- and *Z*- isomers), 7.83 (d, J = 7.1) and 7.79 (d, J = 7.8), (1*H*, H5, *E*- and *Z*- isomers), 7.45-7.25 (6*H*, m, Ph and H3), 7.45-7.25 (m) and 7.19 (d, J = 8.4), (1*H*, NHCH, *E* and *Z*- isomers), 7.10 and 7.08 (1*H*, s, H2, *E*- and *Z*- isomers), 7.00 (1*H*, m, H4), 5.05 and 5.04 (2*H*, s, CH_2_Ph, *E*- and *Z*- isomers), 5.01 and 4.11 (1*H*, m, CH, *E*- and *Z*- isomers), 4.97 (t, J = 5.8) and 4.85 (t, J = 5.6), (1*H*, OH, *E*- and *Z*- isomers 3.86 and 3.84 (3*H*, s, CH_3_, *E*- and *Z*- isomers), 3.80-3.55 (2*H*, m, CH_2_OH). ^13^C NMR (100 MHz, DMSO-d_6_) δ (ppm): 171.2 and 166.7 (COCH, *E*- and *Z*- isomers), 157.7 and 157.6 (C1, *E*- and *Z*- isomers), 155.9 and 155.8 (COO, *E*- and *Z*- isomers), 142.5 and 139.0 (N=CH, *E*- and *Z*- isomers), 137.0 and 136.9 (C6', *E*- and *Z*- isomers), 131.5, 131.3, 128.3, 127.7, 127.6 and 125.4 (C3, C5, C1', C2', C3', C4' and C5'), 122.2, 122.1 and 120.7 (C4 and C6), 111.8 (C2), 65.5 and 65.3 (CH_2_Ph, *E*- and *Z*- isomers), 61.5 and 61.1 (CH_2_OH, *E*- and *Z*- isomers), 56.4 and 54.6 (CH, *E*- and *Z*- isomers), 55.7 (CH_3_). IR (cm^-1^; KBr): 3262 (O—H); 1692 (COCH and COO). EM/ESI: [M+Na]: 370.2.

### Refinement

The C-bound H atoms were geometrically placed (C–H = 0.95–1.00 Å) and refined as riding with *U_iso_*(H) = 1.2-1.5*U_eq_*(parent atom). The O- and N-bound H atoms were refined with the distance restraints 0.84±0.01 and 0.88±0.01 Å, respectively. In the absence of significant anomalous scattering effects, 1278 Friedel pairs were averaged in the final refinement.

### Crystal data

**Table:**

C_19_H_21_N_3_O_5_	*F*(000) = 784
*M**_r_* = 371.39	*D*_x_ = 1.387 Mg m^−^^3^
Orthorhombic, *P*2_1_2_1_2_1_	Synchrotron radiation, λ = 0.6889 Å
Hall symbol: P 2ac 2ab	Cell parameters from 915 reflections
*a* = 6.002 (6) Å	θ = 3.1–23.8°
*b* = 14.053 (14) Å	µ = 0.06 mm^−^^1^
*c* = 21.09 (2) Å	*T* = 120 K
*V* = 1779 (3) Å^3^	Needle, colourless
*Z* = 4	0.30 × 0.04 × 0.02 mm

### Data collection

**Table:**

Rigaku Saturn 724+ detector on Crystal Logics CCD diffractometer	1627 reflections with *I* > 2σ(*I*)
Radiation source: Diamond beamline I19	*R*_int_ = 0.047
silicon double crystal	θ_max_ = 24.3°, θ_min_ = 1.7°
ω scans	*h* = −5→7
13639 measured reflections	*k* = −16→16
1827 independent reflections	*l* = −25→25

### Refinement

**Table:**

Refinement on *F*^2^	Secondary atom site location: difference Fourier map
Least-squares matrix: full	Hydrogen site location: inferred from neighbouring sites
*R*[*F*^2^ > 2σ(*F*^2^)] = 0.030	H atoms treated by a mixture of independent and constrained refinement
*wR*(*F*^2^) = 0.124	*w* = 1/[σ^2^(*F*_o_^2^) + (0.0788*P*)^2^] where *P* = (*F*_o_^2^ + 2*F*_c_^2^)/3
*S* = 1.29	(Δ/σ)_max_ = 0.001
1827 reflections	Δρ_max_ = 0.22 e Å^−^^3^
257 parameters	Δρ_min_ = −0.31 e Å^−^^3^
3 restraints	Absolute structure: nd
Primary atom site location: structure-invariant direct methods	

### Special details

**Table:**

Geometry. All s.u.'s (except the s.u. in the dihedral angle between two l.s. planes) are estimated using the full covariance matrix. The cell s.u.'s are taken into account individually in the estimation of s.u.'s in distances, angles and torsion angles; correlations between s.u.'s in cell parameters are only used when they are defined by crystal symmetry. An approximate (isotropic) treatment of cell s.u.'s is used for estimating s.u.'s involving l.s. planes.
Refinement. Refinement of *F*^2^ against ALL reflections. The weighted *R*-factor wR and goodness of fit *S* are based on *F*^2^, conventional *R*-factors *R* are based on *F*, with *F* set to zero for negative *F*^2^. The threshold expression of *F*^2^ > 2σ(*F*^2^) is used only for calculating *R*-factors(gt) etc. and is not relevant to the choice of reflections for refinement. *R*-factors based on *F*^2^ are statistically about twice as large as those based on *F*, and *R*- factors based on ALL data will be even larger.

### Fractional atomic coordinates and isotropic or equivalent isotropic displacement parameters (Å^2^)

**Table:**

	*x*	*y*	*z*	*U*_iso_*/*U*_eq_	
O1	0.8873 (4)	−0.04357 (14)	0.54989 (10)	0.0315 (5)	
O2	0.8048 (4)	0.11402 (14)	0.55153 (10)	0.0300 (5)	
O3	1.4014 (4)	0.06852 (18)	0.39880 (12)	0.0386 (6)	
H3O	1.531 (3)	0.068 (3)	0.3838 (18)	0.039 (11)*	
O4	0.8614 (4)	0.09631 (14)	0.39964 (10)	0.0281 (5)	
O5	0.8039 (4)	0.54912 (16)	0.29894 (11)	0.0360 (6)	
N1	1.1190 (5)	0.05803 (18)	0.50353 (12)	0.0275 (6)	
H1N	1.161 (6)	0.0066 (16)	0.4822 (15)	0.043 (11)*	
N2	0.9615 (5)	0.25280 (17)	0.39885 (12)	0.0279 (6)	
H2N	1.059 (5)	0.294 (2)	0.4132 (18)	0.054 (12)*	
N3	0.7903 (5)	0.27845 (18)	0.35818 (12)	0.0278 (6)	
C1	0.6559 (6)	−0.1678 (2)	0.59051 (15)	0.0314 (8)	
C2	0.8207 (7)	−0.2227 (2)	0.61849 (16)	0.0369 (8)	
H2	0.9550	−0.1935	0.6322	0.044*	
C3	0.7916 (7)	−0.3195 (2)	0.62669 (17)	0.0404 (9)	
H3	0.9057	−0.3568	0.6456	0.048*	
C4	0.5939 (7)	−0.3618 (2)	0.60700 (16)	0.0415 (9)	
H4	0.5718	−0.4281	0.6132	0.050*	
C5	0.4302 (8)	−0.3084 (3)	0.57863 (18)	0.0443 (9)	
H5	0.2961	−0.3378	0.5649	0.053*	
C6	0.4612 (7)	−0.2106 (2)	0.57000 (17)	0.0384 (9)	
H6	0.3487	−0.1735	0.5501	0.046*	
C7	0.6851 (6)	−0.0622 (2)	0.58501 (16)	0.0333 (8)	
H7A	0.5558	−0.0340	0.5627	0.040*	
H7B	0.6949	−0.0333	0.6277	0.040*	
C8	0.9259 (6)	0.0482 (2)	0.53586 (14)	0.0271 (7)	
C9	1.1576 (5)	0.1469 (2)	0.46987 (14)	0.0256 (7)	
H9	1.1481	0.2004	0.5010	0.031*	
C10	1.3898 (6)	0.1464 (2)	0.44118 (14)	0.0282 (7)	
H10A	1.4175	0.2067	0.4182	0.034*	
H10B	1.5034	0.1397	0.4749	0.034*	
C11	0.9775 (5)	0.1615 (2)	0.41900 (14)	0.0247 (7)	
C12	0.7823 (6)	0.3673 (2)	0.34522 (14)	0.0281 (7)	
H12	0.8937	0.4088	0.3614	0.034*	
C13	0.6034 (6)	0.4056 (2)	0.30579 (14)	0.0290 (7)	
C14	0.6157 (6)	0.4991 (2)	0.28244 (14)	0.0296 (8)	
C15	0.4450 (6)	0.5358 (2)	0.24549 (15)	0.0323 (8)	
H15	0.4531	0.5995	0.2304	0.039*	
C16	0.2632 (7)	0.4798 (2)	0.23060 (15)	0.0339 (8)	
H16	0.1477	0.5046	0.2046	0.041*	
C17	0.2486 (6)	0.3872 (2)	0.25348 (15)	0.0318 (8)	
H17	0.1230	0.3490	0.2434	0.038*	
C18	0.4166 (6)	0.3511 (2)	0.29081 (15)	0.0312 (7)	
H18	0.4050	0.2880	0.3066	0.037*	
C19	0.8395 (7)	0.6391 (2)	0.26835 (16)	0.0374 (9)	
H19A	0.7233	0.6839	0.2815	0.056*	
H19B	0.8337	0.6306	0.2223	0.056*	
H19C	0.9858	0.6641	0.2804	0.056*	

### Atomic displacement parameters (Å^2^)

**Table:**

	*U*^11^	*U*^22^	*U*^33^	*U*^12^	*U*^13^	*U*^23^
O1	0.0299 (14)	0.0250 (10)	0.0396 (12)	0.0018 (10)	0.0069 (11)	0.0038 (9)
O2	0.0292 (14)	0.0283 (11)	0.0326 (11)	0.0038 (10)	0.0030 (10)	−0.0003 (9)
O3	0.0262 (14)	0.0436 (13)	0.0461 (14)	−0.0025 (12)	0.0049 (13)	−0.0140 (11)
O4	0.0239 (14)	0.0278 (11)	0.0327 (11)	−0.0005 (10)	−0.0004 (10)	−0.0024 (9)
O5	0.0347 (14)	0.0299 (11)	0.0434 (13)	−0.0050 (11)	−0.0073 (12)	0.0092 (10)
N1	0.0284 (16)	0.0246 (12)	0.0295 (13)	0.0034 (12)	0.0014 (12)	0.0013 (10)
N2	0.0260 (17)	0.0252 (12)	0.0327 (13)	−0.0009 (12)	−0.0040 (12)	0.0012 (11)
N3	0.0242 (16)	0.0296 (13)	0.0295 (13)	−0.0002 (12)	−0.0019 (12)	0.0016 (10)
C1	0.031 (2)	0.0318 (16)	0.0311 (16)	0.0001 (15)	0.0046 (15)	0.0005 (13)
C2	0.034 (2)	0.0339 (17)	0.0430 (19)	0.0008 (16)	−0.0002 (17)	0.0041 (14)
C3	0.047 (2)	0.0321 (17)	0.0424 (19)	0.0056 (18)	0.0049 (18)	0.0059 (14)
C4	0.055 (3)	0.0294 (15)	0.0401 (18)	−0.0052 (18)	0.015 (2)	−0.0022 (13)
C5	0.044 (3)	0.0455 (19)	0.0435 (19)	−0.0143 (19)	0.0070 (19)	−0.0070 (15)
C6	0.037 (2)	0.0412 (18)	0.0372 (17)	−0.0010 (17)	0.0012 (16)	−0.0022 (15)
C7	0.029 (2)	0.0308 (16)	0.0404 (17)	0.0017 (15)	0.0068 (15)	0.0038 (13)
C8	0.0267 (19)	0.0278 (14)	0.0267 (15)	0.0000 (14)	−0.0030 (14)	0.0017 (12)
C9	0.0244 (18)	0.0243 (14)	0.0280 (15)	0.0013 (13)	0.0010 (14)	0.0007 (11)
C10	0.0267 (19)	0.0255 (14)	0.0324 (16)	−0.0028 (14)	−0.0011 (15)	−0.0005 (12)
C11	0.0203 (18)	0.0259 (14)	0.0279 (15)	−0.0001 (13)	0.0049 (13)	−0.0016 (12)
C12	0.0253 (19)	0.0296 (15)	0.0293 (15)	−0.0038 (14)	−0.0002 (14)	0.0004 (13)
C13	0.031 (2)	0.0296 (15)	0.0265 (15)	0.0030 (15)	−0.0002 (14)	−0.0006 (12)
C14	0.029 (2)	0.0302 (16)	0.0292 (15)	0.0012 (15)	−0.0005 (15)	0.0000 (12)
C15	0.037 (2)	0.0302 (16)	0.0294 (16)	0.0028 (16)	0.0015 (16)	0.0041 (12)
C16	0.034 (2)	0.0364 (18)	0.0309 (17)	0.0069 (16)	−0.0039 (16)	−0.0018 (13)
C17	0.028 (2)	0.0342 (17)	0.0328 (17)	0.0017 (14)	−0.0030 (15)	−0.0018 (13)
C18	0.033 (2)	0.0296 (15)	0.0308 (15)	−0.0004 (16)	−0.0001 (15)	0.0013 (12)
C19	0.044 (2)	0.0275 (16)	0.0410 (18)	−0.0027 (16)	−0.0028 (17)	0.0091 (13)

### Geometric parameters (Å, °)

**Table:**

O1—C8	1.343 (4)	C5—H5	0.9500
O1—C7	1.446 (4)	C6—H6	0.9500
O2—C8	1.222 (4)	C7—H7A	0.9900
O3—C10	1.415 (4)	C7—H7B	0.9900
O3—H3O	0.843 (11)	C9—C10	1.519 (5)
O4—C11	1.221 (4)	C9—C11	1.537 (4)
O5—C14	1.375 (4)	C9—H9	1.0000
O5—C19	1.435 (4)	C10—H10A	0.9900
N1—C8	1.352 (5)	C10—H10B	0.9900
N1—C9	1.455 (4)	C12—C13	1.461 (5)
N1—H1N	0.89 (3)	C12—H12	0.9500
N2—C11	1.355 (4)	C13—C18	1.394 (5)
N2—N3	1.386 (4)	C13—C14	1.405 (4)
N2—H2N	0.88 (3)	C14—C15	1.386 (5)
N3—C12	1.279 (4)	C15—C16	1.382 (5)
C1—C6	1.383 (5)	C15—H15	0.9500
C1—C2	1.386 (5)	C16—C17	1.390 (5)
C1—C7	1.499 (5)	C16—H16	0.9500
C2—C3	1.383 (5)	C17—C18	1.376 (5)
C2—H2	0.9500	C17—H17	0.9500
C3—C4	1.391 (6)	C18—H18	0.9500
C3—H3	0.9500	C19—H19A	0.9800
C4—C5	1.374 (6)	C19—H19B	0.9800
C4—H4	0.9500	C19—H19C	0.9800
C5—C6	1.399 (5)		
			
C8—O1—C7	115.6 (2)	N1—C9—H9	108.5
C10—O3—H3O	107 (3)	C10—C9—H9	108.5
C14—O5—C19	117.3 (3)	C11—C9—H9	108.5
C8—N1—C9	118.1 (3)	O3—C10—C9	107.5 (3)
C8—N1—H1N	115 (3)	O3—C10—H10A	110.2
C9—N1—H1N	114 (2)	C9—C10—H10A	110.2
C11—N2—N3	119.5 (3)	O3—C10—H10B	110.2
C11—N2—H2N	118 (3)	C9—C10—H10B	110.2
N3—N2—H2N	122 (3)	H10A—C10—H10B	108.5
C12—N3—N2	114.4 (3)	O4—C11—N2	124.4 (3)
C6—C1—C2	119.6 (3)	O4—C11—C9	122.3 (3)
C6—C1—C7	120.3 (3)	N2—C11—C9	113.3 (3)
C2—C1—C7	120.1 (3)	N3—C12—C13	120.6 (3)
C3—C2—C1	120.7 (4)	N3—C12—H12	119.7
C3—C2—H2	119.6	C13—C12—H12	119.7
C1—C2—H2	119.6	C18—C13—C14	118.5 (3)
C2—C3—C4	119.4 (4)	C18—C13—C12	121.2 (3)
C2—C3—H3	120.3	C14—C13—C12	120.3 (3)
C4—C3—H3	120.3	O5—C14—C15	124.0 (3)
C5—C4—C3	120.4 (3)	O5—C14—C13	115.6 (3)
C5—C4—H4	119.8	C15—C14—C13	120.4 (3)
C3—C4—H4	119.8	C16—C15—C14	119.9 (3)
C4—C5—C6	119.9 (4)	C16—C15—H15	120.0
C4—C5—H5	120.0	C14—C15—H15	120.0
C6—C5—H5	120.0	C15—C16—C17	120.3 (3)
C1—C6—C5	119.9 (4)	C15—C16—H16	119.9
C1—C6—H6	120.1	C17—C16—H16	119.9
C5—C6—H6	120.1	C18—C17—C16	119.8 (3)
O1—C7—C1	108.5 (3)	C18—C17—H17	120.1
O1—C7—H7A	110.0	C16—C17—H17	120.1
C1—C7—H7A	110.0	C17—C18—C13	121.1 (3)
O1—C7—H7B	110.0	C17—C18—H18	119.5
C1—C7—H7B	110.0	C13—C18—H18	119.5
H7A—C7—H7B	108.4	O5—C19—H19A	109.5
O2—C8—O1	124.4 (3)	O5—C19—H19B	109.5
O2—C8—N1	124.7 (3)	H19A—C19—H19B	109.5
O1—C8—N1	110.9 (3)	O5—C19—H19C	109.5
N1—C9—C10	109.7 (3)	H19A—C19—H19C	109.5
N1—C9—C11	110.1 (3)	H19B—C19—H19C	109.5
C10—C9—C11	111.6 (2)		
			
C11—N2—N3—C12	175.9 (3)	N3—N2—C11—C9	−173.0 (2)
C6—C1—C2—C3	−0.6 (5)	N1—C9—C11—O4	−18.0 (4)
C7—C1—C2—C3	176.8 (3)	C10—C9—C11—O4	104.0 (3)
C1—C2—C3—C4	−0.5 (5)	N1—C9—C11—N2	161.6 (3)
C2—C3—C4—C5	1.2 (5)	C10—C9—C11—N2	−76.3 (3)
C3—C4—C5—C6	−0.7 (5)	N2—N3—C12—C13	−176.6 (3)
C2—C1—C6—C5	1.1 (5)	N3—C12—C13—C18	11.7 (5)
C7—C1—C6—C5	−176.3 (3)	N3—C12—C13—C14	−168.8 (3)
C4—C5—C6—C1	−0.5 (5)	C19—O5—C14—C15	−9.5 (5)
C8—O1—C7—C1	174.1 (3)	C19—O5—C14—C13	170.7 (3)
C6—C1—C7—O1	−124.9 (3)	C18—C13—C14—O5	180.0 (3)
C2—C1—C7—O1	57.7 (4)	C12—C13—C14—O5	0.4 (4)
C7—O1—C8—O2	1.1 (4)	C18—C13—C14—C15	0.1 (5)
C7—O1—C8—N1	179.6 (3)	C12—C13—C14—C15	−179.4 (3)
C9—N1—C8—O2	−17.9 (4)	O5—C14—C15—C16	179.1 (3)
C9—N1—C8—O1	163.6 (3)	C13—C14—C15—C16	−1.1 (5)
C8—N1—C9—C10	175.6 (3)	C14—C15—C16—C17	1.2 (5)
C8—N1—C9—C11	−61.2 (3)	C15—C16—C17—C18	−0.4 (5)
N1—C9—C10—O3	60.0 (3)	C16—C17—C18—C13	−0.6 (5)
C11—C9—C10—O3	−62.3 (3)	C14—C13—C18—C17	0.7 (5)
N3—N2—C11—O4	6.6 (5)	C12—C13—C18—C17	−179.8 (3)

### Hydrogen-bond geometry (Å, °)

**Table:**

*D*—H···*A*	*D*—H	H···*A*	*D*···*A*	*D*—H···*A*
N1—H1n···O3	0.89 (3)	2.44 (3)	2.788 (5)	104 (2)
O3—H3o···O4^i^	0.85 (2)	2.04 (2)	2.789 (4)	147 (4)
N2—H2n···O2^ii^	0.88 (3)	2.10 (3)	2.974 (5)	177 (3)
C10—H10b···O2^i^	0.99	2.45	3.439 (5)	175
C16—H16···O4^iii^	0.95	2.55	3.284 (5)	134

Symmetry codes:  (i) *x*+1, *y*, *z*; (ii) *x*+1/2, −*y*+1/2, −*z*+1; (iii) −*x*+1, *y*+1/2, −*z*+1/2.
